# A Novel Technique for Occipitocervical Fusion with Triple Rod Connection to Prevent Implant Failure

**DOI:** 10.7759/cureus.24821

**Published:** 2022-05-08

**Authors:** Fumihiko Eto, Hiroshi Takahashi, Toru Funayama, Masao Koda, Masashi Yamazaki

**Affiliations:** 1 Department of Orthopaedic Surgery, Faculty of Medicine, University of Tsukuba, Tsukuba, JPN

**Keywords:** rheumatoid arthritis, athetoid cerebral palsy, additional rod, implant failure, occipitocervical fusion

## Abstract

Occipitocervical fusion is an effective surgical method for treating various upper cervical disorders. However, complications such as implant failure due to rod breakage have been reported. Therefore, we devised a surgical technique for occipitocervical fusion with a triple rod connection to prevent implant failure. Occipitocervical fusion with triple rod connection was performed in two cases with a high risk of instability such as athetoid cerebral palsy and rheumatoid arthritis. A multiaxial screw (diameter: 4.5 mm) was inserted into the screw hole in the middle of the occipital plate, and subsequently, an additional rod was attached. It was connected to the main rod using an offset connector at the caudal side. The connection of the additional rod was simple and did not interfere with the fusion bed for bone graft between the occipital bone and axis. The head of the screw was crimped to the occipital plate, and the plate was firmly fixed. Moreover, since the head of the screw did not protrude to the dorsal side, the tension of the soft tissue and skin did not increase. No complications occurred after surgery in both cases. In addition, no special instruments were required to connect the additional rod to the main rod in this procedure. Therefore, our technique may be useful as an option to prevent implant failure due to rod breakage at the craniocervical junction.

## Introduction

Occipitocervical fusion is an effective surgical method for treating various upper cervical pathologies such as degenerative, traumatic, neoplastic, congenital, and infective lesions [1,2]. Previously, wiring in combination with bone grafting was commonly performed. However, this method usually requires long-term halo-vest fixation, which is associated with a high rate of complications, such as loosening of pins, infection, pneumonia, and dysphagia [3]. To avoid wearing a halo vest after surgery, it is desirable to perform strong internal fixation during occipitocervical fusion. Therefore, procedures using occipital plates, cervical screws, and spinal rods are currently popular [1]. However, cervical lesions in patients with athetoid cerebral palsy (CP) or rheumatoid arthritis (RA) are extremely unstable and have a high risk of implant failure due to rod breakage at the craniocervical junction [4,5]. Nevertheless, there is no established surgical technique to reinforce the implant to prevent failure. Therefore, we devised a surgical technique to prevent implant failure after occipitocervical fusion by achieving mechanical stability using three spinal rods.

## Case presentation

Surgical technique of occipitocervical fusion with triple rod connection

**Figure 1:**
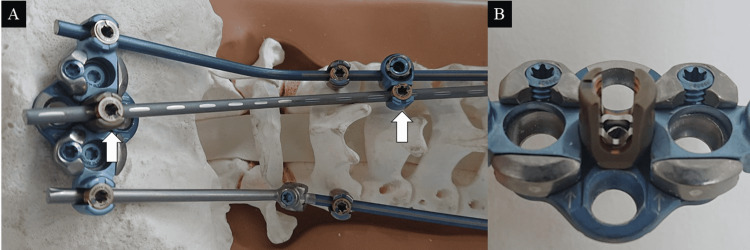
Surgical technique of occipitocervical fusion with triple rod connection (A) Bone model of occipitocervical fusion with triple rod connection. An additional rod is placed between the main rods (arrows). (B) A multiaxial screw (diameter: 4.5 mm) is inserted into the middle hole of the occipital plate.

The instrument used was VuePoint Ⅱ (NuVasive, Inc., San Diego, CA). Depending on the case, pedicle, laminar, lateral mass, and paravertebral foremen screws were used [6]. An occipital plate with three holes in the midline and two holes in the paramedian line was placed on the occipital bone. A multiaxial (MA) screw (diameter: 4.5 mm) was inserted into the central screw hole of three median holes of the occipital plate, and a cortical screw (diameter: 4.5 mm) was inserted into the other holes. Occipital bone, C2 spinous process, and vertebral arch were decorticated, and tricortical bone block and cancellous bone from the ilium were grafted before installing the additional rod. After installing titanium alloy main rods (3.5 mm diameter for each) on both sides, an additional rod was attached to bridge the craniocervical junction from the head of the screw inserted in the middle of the occipital plate, and the caudal side was connected to the main rod using an offset connector (Figure 1).

Occipitocervical fusion with triple rod connection was performed in two patients with severe instability of the upper cervical spine after confirming that the placement of the additional rod could be easily performed using model bone (Table 1). The patients provided written informed consent to use clinical and imaging data and intraoperative photographs for publication and the off-label usage of the screw.

**Table 1:** Data of the patients in whom occipitocervical fusion with triple rod connection was performed

Case	Age (year)	Sex	Diagnosis	Fusion level of primary surgery	Type of failure	Onset of failure (months)
1	61	Male	Athetoid cerebral palsy	C1-T1	Bilateral screw breakages (C1)	6
2	68	Female	Rheumatoid arthritis	Oc-C7	Unilateral rod breakage	6

Case 1

**Figure 2:**
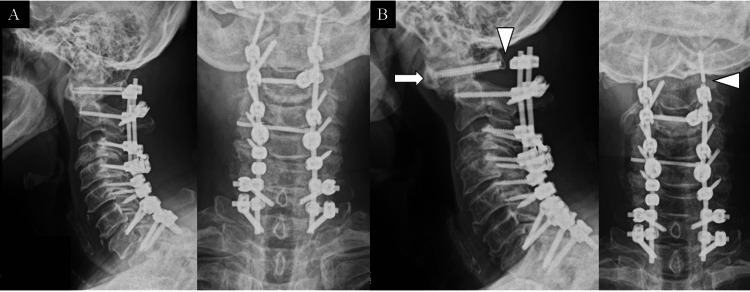
Case 1 (A) X-ray image after primary surgery (C1-T1 fusion). (B) X-ray image obtained six months after the primary surgery shows bilateral C1 screw breakages (arrowhead) and recurrence of atlantoaxial subluxation (arrow).

**Figure 3:**
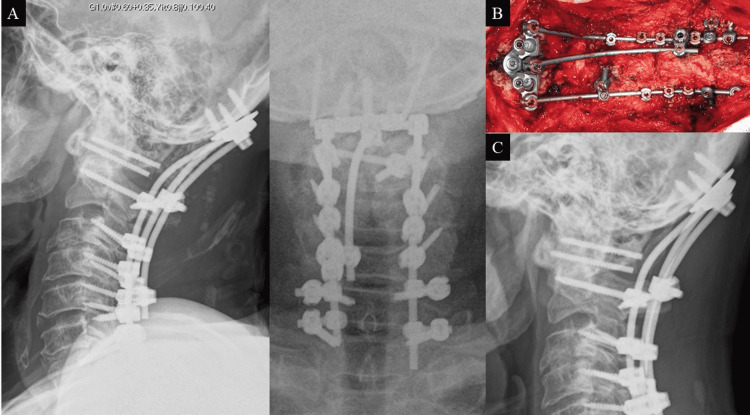
Revision surgery in case 1 (A) X-ray image after Oc-T1 fusion with triple rod connection. (B) Intraoperative photograph obtained before bone grafting demonstrates the triple rod connection. (C) X-ray image obtained 12 months after revision surgery shows bony fusion without implant failure.

A 61-year-old man with athetoid CP visited our hospital with complaints of clumsiness of both hands and gait disturbance that worsened over several months. The Japanese Orthopaedic Association (JOA) score was 4 points. Posterior cervicothoracic fusion (C1-T1) and decompression (C3/4) were performed for myelopathy with the instability of C1/2 and C3/4. Three months after the operation, finger movements improved, and gait supported with a walker became possible (JOA score: 10.5 points). However, six months after the operation, neck pain, clumsiness of both hands, and gait disturbance worsened again (JOA score: 8.5 points). Bilateral C1 screw breakages were observed, and the recurrence of myelopathy due to atlantoaxial subluxation (AAS) was diagnosed (Figure 2). Posterior decompression (C1) and occipitocervicothoracic fusion (Oc-T1) with triple rod connection were performed as revision surgery (Figures 3A, 3B). After the operation, a halo vest was worn for two months to prevent re-failure. Therefore, bony fusion was obtained 12 months after revision surgery without implant failure (Figure 3C). There was no neck pain, and clumsiness of both hands improved (JOA score: 9.5 points).

Case 2

**Figure 4:**
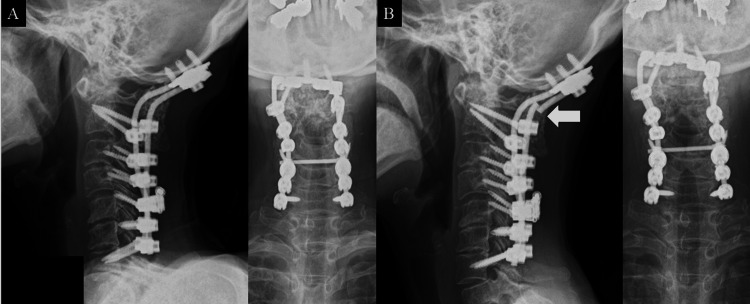
Case 2 (A) X-ray image after primary surgery (Oc-C7 fusion). (B) X-ray image obtained six months after the primary surgery shows unilateral rod breakage (arrow).

**Figure 5:**
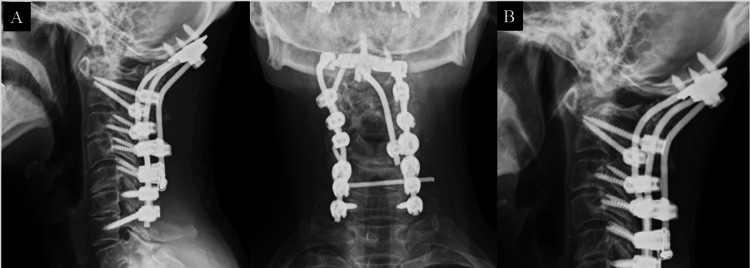
Revision surgery in case 2 (A) X-ray image after rod exchange with triple rod connection. (B) X-ray image obtained six months after revision surgery shows bony fusion without implant failure.

A 68-year-old woman with a history of RA for 25 years and osteoporosis (young adult mean: 62%) was referred to our hospital for limb weakness and gait disturbance that had worsened over several months (JOA score: −0.5 points). Myelopathy with not only AAS but also spinal instability and cord compression at C3/4 was diagnosed, and posterior decompression (C1 and C3/4) and occipitocervical fusion (Oc-C7) were performed (Figure 4A). After the operation, her limb strength and gait disturbance improved (JOA score: 11 points). However, six months after the operation, asymptomatic unilateral rod breakage was observed at the craniocervical junction (Figure 4B). Rod exchange with triple rod connection was performed as revision surgery (Figure 5A). After the operation, a soft collar was worn for a month. Bony fusion was obtained six months after revision surgery without implant failure (Figure 5B), and symptoms were unchanged from before revision surgery (JOA score: 11 points).

**Figure 6:**
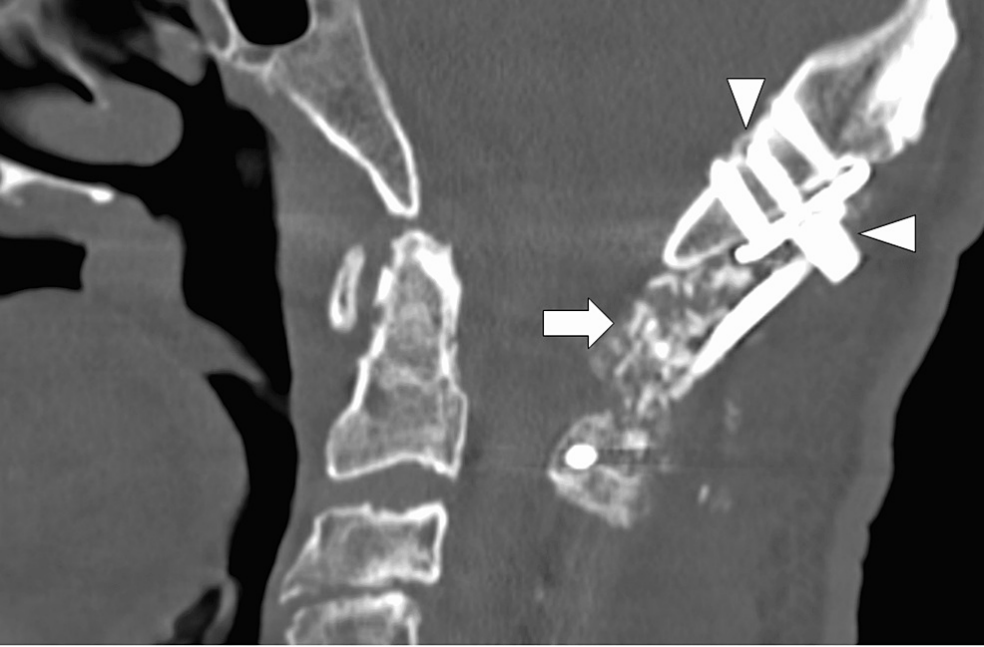
Postoperative computed tomography in case 1 A multiaxial screw is inserted into the contralateral cortex, and the head of the screw is crimped to the plate (arrowheads). Bone grafting is not interfered with by the additional rod (arrow).

In both cases, the additional rod was connected in the midline; therefore, the paraspinal muscle detachment could be limited to the extent necessary to place the occipital plate and cervical screws. The head of the MA screw was crimped to the occipital plate; therefore, the occipital plate was firmly fixed. The additional rod was easy to connect and did not interfere with the fusion bed of the bone graft between the occipital bone and axis (Figure 6). Furthermore, since the head of the screw did not protrude to the dorsal side, the tension of the soft tissue and skin did not increase during wound closure. No complications occurred after surgery in both cases.

## Discussion

We devised a novel triple rod connection technique for implant reinforcement during occipitocervical fusion. In this study, the procedure was performed as revision surgery. However, it may be also useful as a primary surgery for upper cervical lesions with a high risk of instability.

In cervical posterior instrumentation surgery, thick titanium rods with 4.0 mm diameter or cobalt-chromium rods were used to increase fixation strength. However, the devices with 4.0 mm diameter titanium rods are limited. Furthermore, it has been reported that junctional kyphosis may occur more frequently with cobalt-chromium systems than with titanium alloy systems after multiple intervertebral fusions [7]. Therefore, in occipitocervical fusion, since the stress on the distal junction of the fixation increases, the risk of adjacent segmental disorder such as vertebral fracture is a concern. Additional sublaminar wiring, another technique to increase fixation strength, can increase pullout strength but cannot prevent rod breakage. Therefore, further innovations in the reinforcement of spinal rods are needed.

In recent years, a procedure using multi-rod constructs has been reported to prevent rod breakage in long spinal fusion for adult spinal deformity [8]. Because rod breakage at the lumbosacral junction is caused by mechanical instability, reinforcement with an additional rod is considered beneficial. Therefore, we believed that it would be possible to prevent rod breakage by reinforcing the spinal rod with an additional rod in the craniocervical junction as well. We devised the triple rod connection technique to reinforce the implant by inserting a 4.5 mm diameter MA screw into the screw hole of the occipital plate and placing an additional rod in the midline. Using an offset connector on the caudal side made it possible to easily connect to the main rod without using special instruments. Moreover, because only the intervertebral segment that needs to be firmly fixed can be reinforced with an additional rod, we considered reducing the stress on the distal junction compared with using main rods of cobalt-chromium. Liu et al. reported a procedure using a spinous process screw in C2 as a third anchor for occipitocervical fusion [9]. However, in this report, there were concerns about the effect on the fusion bed of the bone graft and the penetration of the screw into the spinal canal. Conversely, our technique is safer because there is no possibility of bone graft affecting the fusion bed or penetration of the screw into the spinal canal.

If an additional rod is to be connected to the exterior of the main rod during occipitocervical fusion, the invasion of soft tissue may be increased because of the widening of the range of detachment of paraspinal muscles. In our procedure, of the three screws in the middle of the occipital plate, the longest screw could have been inserted into the most cranial side with a thick bone cortex. However, the head of the screw might protrude to the dorsal side, causing skin and soft tissue problems. Therefore, the MA screw was inserted into the central screw hole. In both cases, a screw with a length of 10 mm or more could be inserted, and the occipital plate was firmly fixed. Since the additional rod was placed in the middle and had a low profile, this procedure was considered useful for older patients or patients with RA who had fragile soft tissue and skin.

This study has several limitations. First, there are no biomechanical data to demonstrate the superiority of the mechanical strength of the construct in this technique compared to that in the conventional method; therefore, it will be investigated in the future study. Second, the instruments used in this study had good compatibility with the MA screw and occipital plate. However, it is necessary to investigate whether this procedure can be performed using other instruments. Third, the length of the MA screw inserted into the occipital bone was the shortest at 10 mm. However, there may be patients in whom this procedure cannot be performed because of the risk of protrusion of the screw into the skull depending on the shape of the occipital bone. Therefore, it is important to create an appropriate preoperative plan using computed tomography.

## Conclusions

Occipitocervical fusion with triple rod connection was performed for upper cervical lesions with a high risk of instability. In this procedure, no special instruments were required to connect the additional rod to the main rod without affecting the fusion bed of the bone graft, and no adverse events such as skin or soft tissue problems occurred. Therefore, this technique may be useful as an option for both primary and revision surgery to prevent implant failure due to rod breakage at the craniocervical junction.
